# Thoracolumbar flexion dysfunction and thoracolumbar compression fracture in postmenopausal women: a single-center retrospective study

**DOI:** 10.1186/s13018-021-02857-w

**Published:** 2021-12-07

**Authors:** Zhirong Zheng, Chao Liu, Zhen Zhang, Wenhao Hu, Meng Gao, Chengqi Jia, Xuesong Zhang

**Affiliations:** 1 Medical School of Chinese PLA, Beijing, China; 2 Department of Orthopedics, The First Medical Centre, Chinese PLA General Hospital, 28 Fuxing Road, Haidian District, Beijing, China; 3 Department of Orthopedics, The Fourth Medical Centre, Chinese PLA General Hospital, Beijing, China

**Keywords:** Thoracolumbar flexion dysfunction, Vertebral fracture, Osteoporosis, Postmenopausal women, Quantitative computed tomography

## Abstract

**Objective:**

To investigate whether thoracolumbar flexion dysfunctions increase the risk of thoracolumbar compression fractures in postmenopausal women.

**Methods:**

The records of postmenopausal women with thoracolumbar vertebral compression fractures and without vertebral compression fractures were surveyed. Demographic data, clinical data, and quantitative computed tomography (QCT) findings were compared between the groups. Chi-squared tests, unpaired *t*-tests, Spearman, and Mann–Whitney U were used to assess the group characteristics and proportions. The relationship between the risk of fracture and the difference of Cobb’s angle of thoracolumbar segment (DCTL) was evaluated by logistic regression. DCTL was calculated by subtracting thoracolumbar Cobb’s angles (TLCobb’s) from thoracolumbar hyperflexion Cobb’s angles (TLHCobb’s). Quantitative computed tomography (QCT) values and spinal osteoarthritis (OA) of postmenopausal women in the two groups were compared.

**Results:**

102 of 312 were enrolled to the study group of postmenopausal women with the fracture, and 210 of 312 were enrolled to the control group of postmenopausal women without the fracture. There were significant differences in QCT values and spinal OA including disc narrowing (DSN) and osteophytes (OPH) between the two groups (*p* < 0.001 for all four). The risk of thoracolumbar compression fractures in the postmenopausal women with DCTL ≤ 8.7° was 9.95 times higher (95% CI 5.31–18.64) than that with > 8.7° after adjusting for age, BMI, and QCT values.

**Conclusion:**

Low DCTL may be a risk factor of thoracolumbar compression fractures in postmenopausal women, and a DCTL ≤ 8.7° can be a threshold value of thoracolumbar compression fractures.

## Introduction

Osteoporotic fracture is a common complication that imposes an enormous medical, psychological, social, and economic cost on individuals, families, and society [1–4]. With appropriate screening, healthcare providers can implement effective interventions before fractures occur and ultimately improve quality of life, as well as help curb tremendous osteoporosis-related costs. On the other hand, thoracolumbar spine, especially on the range from T11 to L2, has the highest incidence of osteoporotic fracture, on which there are most of the fractures in postmenopausal women [4–6]. Thus, it may be cost-effective to screen from the thoracolumbar spine in postmenopausal women.

Thoracolumbar flexion dysfunction, as a dysfunction of the center connecting thoracic spine and lumbar spine, plays a prominent role in spinal dysfunction [7]. People's work and daily life are usually inseparable from the flexion of the spine, especially forward flexion, such as picking up an apple on the ground. Once there is spinal flexion dysfunction, the body will become stiff, which will not only affect the movement ability, but also affect the cushioning capacity against external impact, such as spinal fracture in patients with ankylosing spondylitis [8]. Degeneration of intervertebral disc may be a precipitating risk factor for spinal flexion dysfunctions, while intervertebral disc joins in limiting the range of spinal motion of flexion and extension [9–11]. With aging, changes in the tissue properties of the intervertebral disc, including dehydration and reorganization of the nucleus pulposus and fragmentation and stiffening of the annulus fibrosus, markedly alter the mechanics of load transfer in the spine [12–15]. The degeneration of intervertebral disc may be one of the contributors of the relationship suggested by Schneider, D.L.et al. that disc narrowing is significantly associated with an increased risk of vertebral fracture [16]. Thus, in the process of increasing the risk of vertebral compression fracture, the degeneration of intervertebral disc may also contribute to the formation of spinal flexion dysfunction.

**Fig. 1:**
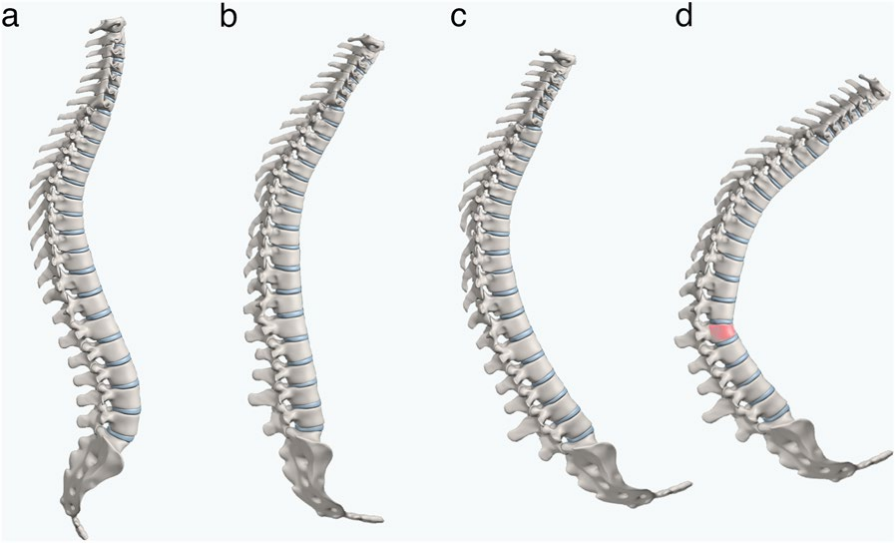
Neutral spinal posture is in static equilibrium (**a**). Dynamic flexion postures (**a**–**d**) with low-speed compression show the process of low-energy vertebral fracture

It has been accepted that flexion posture is more common in low-energy spinal injuries than neutral posture [17, 18]. In most low-energy injuries, such as fall, the spine tends to move from neutral posture to flexion posture or hyperflexion posture because of the joint action of upper body, hip, and the center of gravity (Fig. 1) [18]. In addition, thoracolumbar spine, as a junction of lumbar vertebrae and thoracic vertebrae, is a weak position of mechanical structure [19, 20]. As a result, vertebrae from T11 to L2 have the highest incidence of deformity and fracture in postmenopausal women [21–23]. In a case of low-energy fall, the spinal transition from neutral posture to hyperflexion posture may induce the increased risk of compression fractures at the vertebrae from T11 to L2. Therefore, thoracolumbar hyperflexion position in X-ray, as a same posture of low-energy spinal injuries, may provide a way of investigating the risk of thoracolumbar vertebral compression fracture.

The purpose of the present study was to evaluate the difference of Cobb’s angle of thoracolumbar segment (DCTL) from thoracolumbar hyperflexion Cobb’s angle (TLHCobb’s) to thoracolumbar neutral Cobb’s angle (TLCobb’s), and to ascertain the relationship between the risk of thoracolumbar compression fractures and DCTL, as well as determine a possible clinic threshold value of DCTL.

## Materials and methods

This study protocol was approved by the Human Ethics Committee of Chinese PLA General Hospital. All participants were informed about the study, and they signed a written consent for inclusion.

**Fig. 2:**
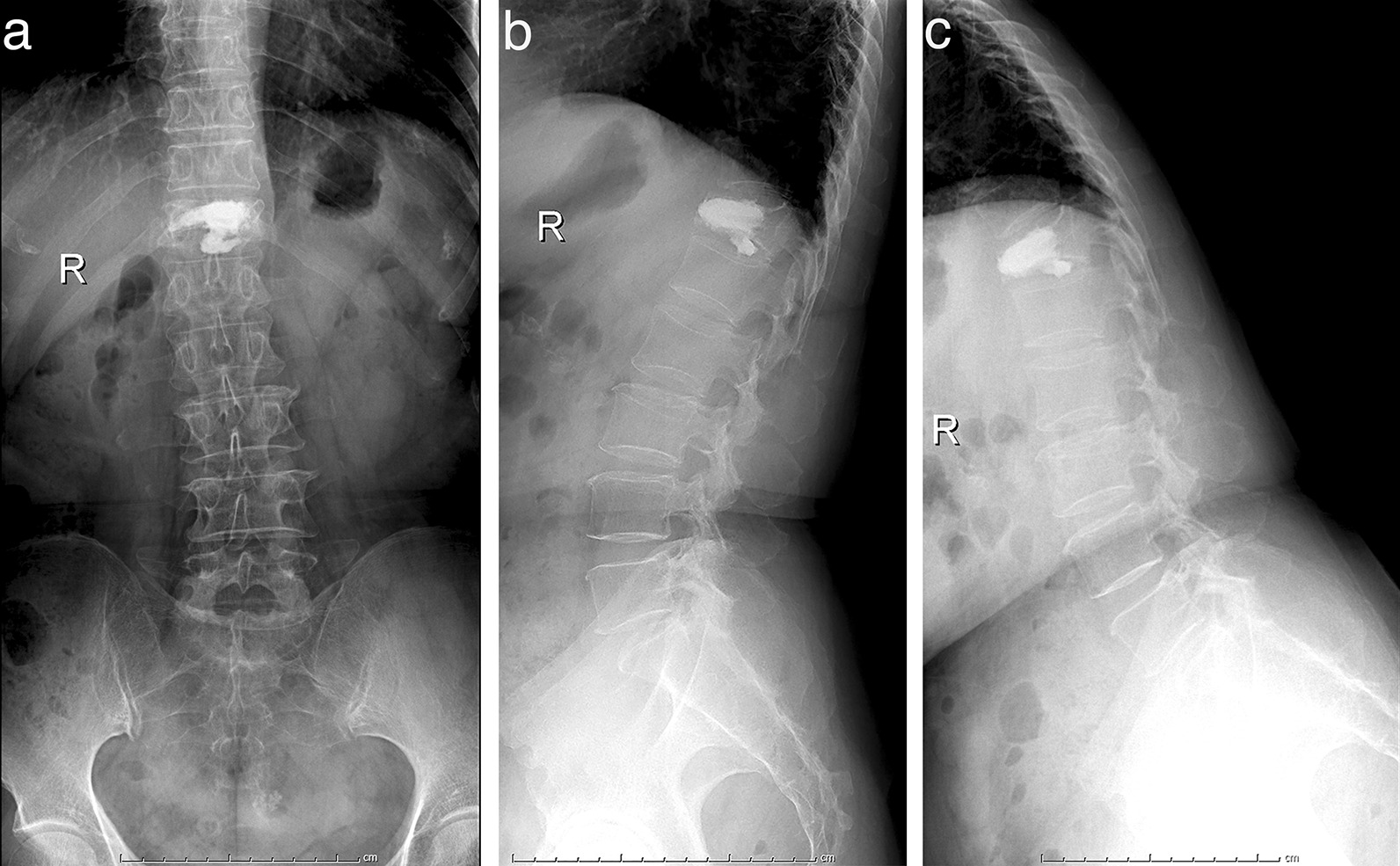
The radiographic data included lumbar anteroposterior (**a**), lateral (**b**) and lateral hyperflexion (**c**) lumbar X-rays

This study was a single-center retrospective study. The records of postmenopausal women treated at Chinese PLA General Hospital from July 2018 to June 2020 were surveyed. The study group inclusion criteria were (1) one-level osteoporotic vertebral compression fracture situated from T11 to L2, (2) treatment with vertebroplasty (3) complete radiographic data including lateral hyperflexion lumbar X-ray (Fig. 2). The exclusion criteria were (1) lumbar disc herniation, spinal instability and spinal spondylolisthesis, (2) spinal tumors, (3) spinal scoliosis (coronal Cobb’ angle > 10°), and (4) a previous history of osteoporotic fracture or vertebral augmentation operation. The control group was age-matched (unpaired *t*-test, *p* > 0.05) postmenopausal women with healthy spine, excluding (1) lumbar disc herniation, spinal instability and spinal spondylolisthesis, (2) spinal tumors, ankylosing spondylitis, inflammatory spondylitis, congenital spinal deformity, spinal scoliosis (Cobb’s angle > 10°), and (3) a previous history of osteoporotic fracture (4) incomplete radiographic data.

Age, height, weight, body mass index (BMI; kg/m^2^), and other information of the two groups were evaluated. Posterior-anterior, lateral, and hyperflexion radiographic data of lumbar spine were collected. Data on fracture site, Cobb’s angle, and vertebral QCT value from T11 to L2 were recorded.

**Fig. 3:**
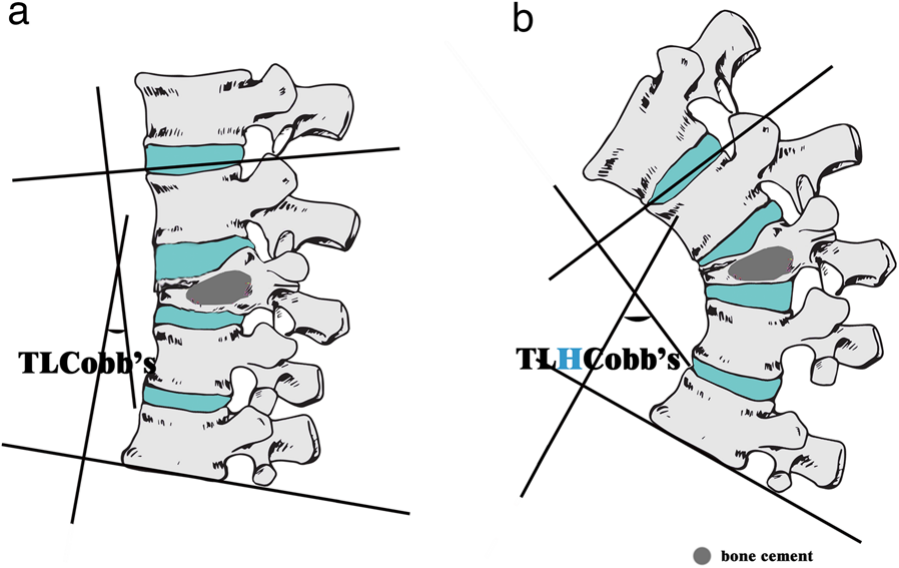
DCTL was the difference of the Cobb’s angle from T11 to L2 between hyperflexion position (**b**) and neutral position (**a**) in lateral X-ray

On a lateral X-ray, TLHCobb’s and TLCobb’s were measured by drawing two lines, respectively, through the superior endplate of T11 and the inferior endplate of L2, respectively, on the hyperflexion and neutral positions of lumbar spine (Fig. 3). The Cobb’s angle was defined as “ + ”, while spine was kyphosis, and “ -” while lordosis, accordingly. The TLCobb’s in study group was assessed by excluding the contributions of fracture-and-operation of fractured vertebra according to Tian's suggestion, as following [24].

**Fig. 4:**
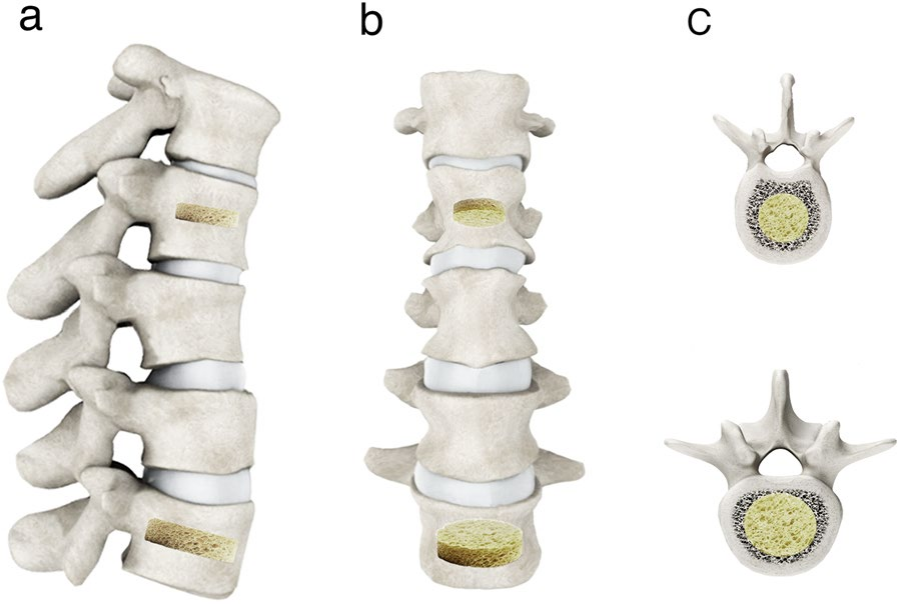
The region of interest (ROI) in this study was defined as the largest volume of oval cylinder in the middle of vertebral body in sagittal (**a**), coronal (**b**) and axial (**c**) positions to reduce the effect of hyperplasia and sclerosis in vertebral body.

**Fig. 5:**
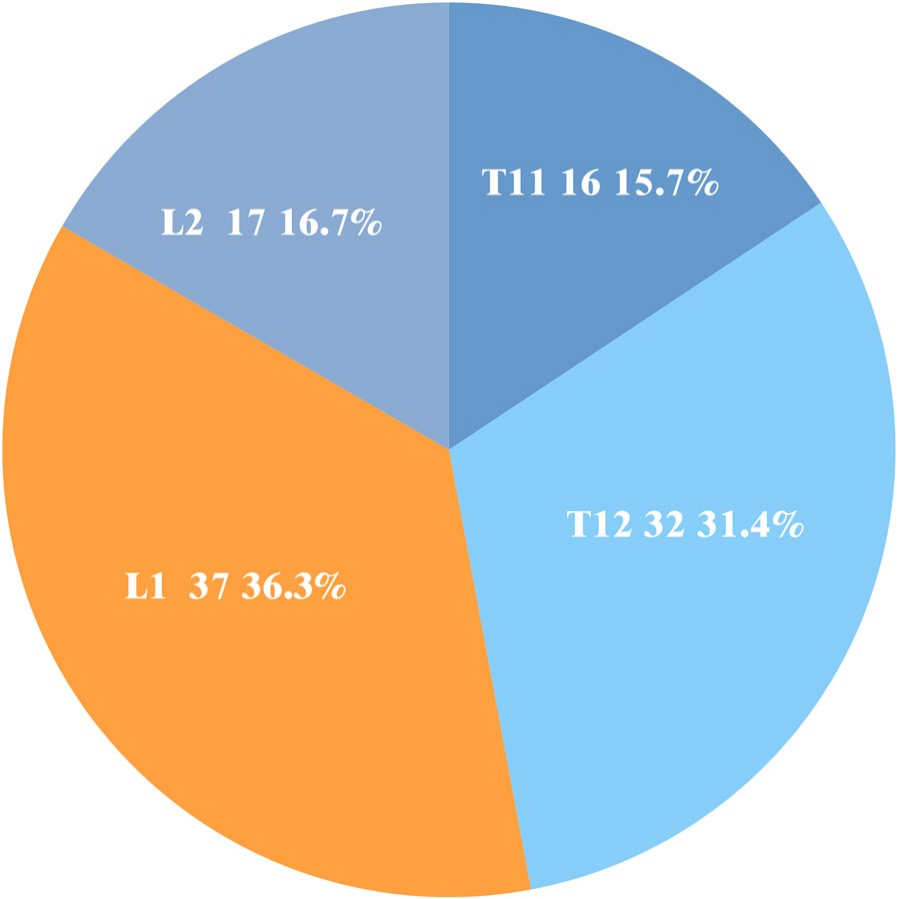
The study group consisted of postmenopausal patients with vertebral compression fracture at T11 in 16 (15.6%), T12 in 32 (31.3%), L1 in 37 (36.5%), and L2 in 17 (16.7%)

**Fig. 6:**
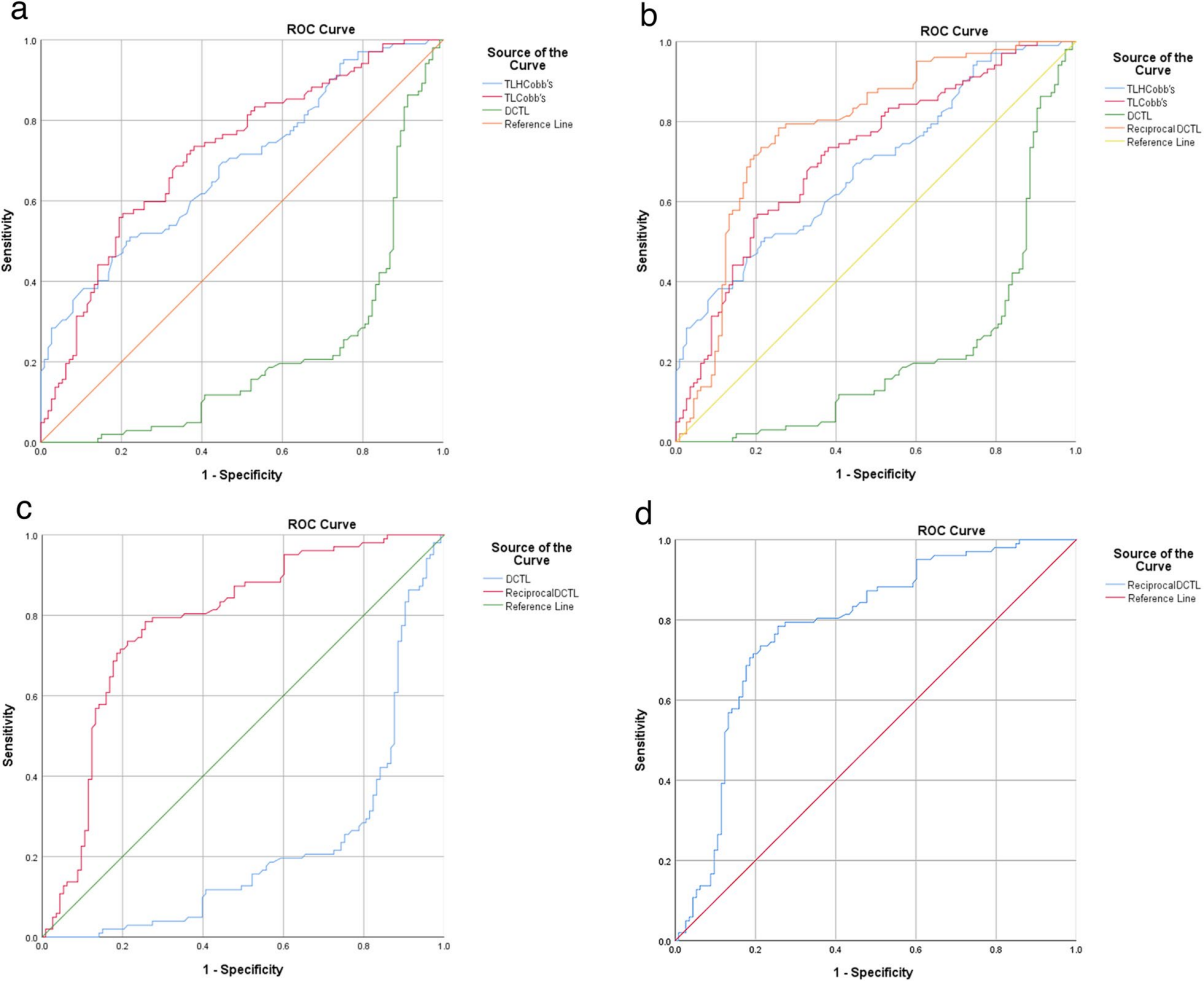
Receiver operating characteristic (ROC) analyses. ROC analysis to identify the threshold value of DCTL, TLHCobb’s and TLCobb’s. DCTLs of 8.7°, 7.5° and 9.2° were determined using the ROC curve of reciprocal DCTL and Youden’s index. The three lines showed the differences among DCTL, TLHCobb’s and TLCobb’s (**a**). Reciprocal DCTL line presented the higher accuracy of detection than TLHCobb’s and TLCobb’s lines (**b**, **c** and **d**), and a threshold value of 8.7° with a sensitivity of 78. 4%, a specificity of 74.3%, and an AUC of 0.783 (95% CI of 0.613–0.953)

TLCobb’s = postoperative TLCobb’s − (postoperative Cobb’s angle of fractured vertebra − mean value of the Cobb’s angle of non-fractured vertebrae at the same level of fractured vertebra in the study group).

DCTL was calculated by subtracting TLCobb’s from TLHCobb’s.

The maximal value of cancellous region was drawn in the middle of the vertebral body (Fig. 4). The mean value of QCT of two vertebrae L1 and L2 was calculated [25]. QCT value of fractured vertebrae was obtained from calculating the half of the sum of QCT value of the two adjacent vertebrae of the fractured vertebra, according to Soliman’s suggestion [26]. BMD was classified as normal (BMD > 120 mg/cm^3^), osteopenia (80–120 mg/cm^3^), and osteoporosis (BMD < 80 mg/cm^3^) according to the criteria of the World Health Organization and the American College of Radiology (ACR). BMD was measured using a QCTPRO2.0 workstation (Mindways Software Inc., Austin, TX, USA) and quantitative computed tomography (QCT) scans in a 16-slice spiral CT scanner (GE Discovery CT750 HD) with the following parameters: 120 kV, 125 mA, pitch 0.985, SFOV 500 mm, aperture 1.25 mm, and table height 780 mm.

On the lateral radiographs, spinal osteoarthritis (OA) including disc narrowing (DSN) and osteophytes (OPH) was used to evaluate lumbar degeneration according to the suggestion of Lane, Sornay-Rendu et al. [16, 27]. Then, fourpoint scale: normal (0), mild (1), moderate (2), or severe (3) was used to evaluate DSN and OPH. Spinal osteoarthritis (OA) was defined as Grade 0 if both scores were normal; the Grade 1 with scores mild OPH or DSN; the Grade 2 with scores moderate or severe DSN or OPH. The interobserver reproducibility [k (95% CI)] assessed on radiographs by two trained radiologist was 0.85 (95% CI 0.68–0.96) and 0.68 (95% CI 0.39–0.89) at the lumbar spine for DSN and OPH scores, respectively.

For statistical analyses, data were analyzed using SPSS version 26 (IBM, Chicago, IL, USA). All variables were tested for normal distribution with the Kolmogorov–Smirnov test, and data were considered normally distributed if *p* > 0.05. The total data showed a nonnormal distribution (*p* < 0.05). Descriptive statistics for continuous variables were expressed as means and standard deviations. Differences in age, height, BMI, QCT values, and Cobb’s angles between the two groups were assessed using Chi-squared and unpaired *t*-tests. Differences in spinal OA, DSN, and OPH between the two groups were assessed with Mann–Whitney U. Correlation analysis between DSN and DCTL was assessed with Spearman. Statistical tests for trend by increasing number of prevalent fractures were performed by including an ordinal variable for mild, moderate, and severe DCTLs, in regression models. Association between number of prevalent fractures and DCTLs was evaluated by including an reciprocal value to DCTL in the logistic regression model. Odds ratios (ORs) with 95% confidence intervals (CIs) were calculated after adjusting for age, BMI, and QCT values. The threshold value of DCTL was determined by a receiver operating characteristic (ROC) curve and Youden’s index.

## Results

A total of 115 postmenopausal women with vertebral compression fracture were selected according to the inclusion criteria, of which 102 postmenopausal women were included in the study group per the exclusion criteria. There were 16 T11 (15.6%), 32 T12 (31.3%), 37 L1 (36.5%), and 17 L2 (16.7%) compression fractures (Fig. 5). Of the 234 postmenopausal women without vertebral compression fractures, 210 postmenopausal women with healthy spine were included in the control group according to the exclusion criteria.

Despite the lack of differences in age, height, BMI, and the average vertebra Cobb’s angle in T11–L2 between the two groups (Table 1, *p* > 0.05), the QCT values in the study group were lower than those in the control group (Table 2, *p* < 0.001).

**Table 1:** Demographic and clinic Characteristics of postmenopausal women in study and control group (n = 312)

Variables	Study group (n = 102)	Control group (n = 210)	p
Age (year) Height (cm) BMI (kg/m^2^)	66.47 ± 6.11 156.45 ± 8.24 25.11 ± 2.48	66.21 ± 6.98 159.23 ± 3.94 24.90 ± 2.37	0.219 0.534 0.403
Vertebral Cobb’s angles			
T11 T12 L1 L2 Coronal TLCobb’s	5.51 ± 2.59° (86) 5.88 ± 2.33° (70) 5.85 ± 2.18° (65) 4.24 ± 2.33° (85) 1.82 ± 2.25	5.17 ± 2.55° 5.41 ± 2.27° 5.27 ± 2.36° 3.97 ± 2.22° 1.66 ± 2.37	0.414 0.110 0.055 0.213 0.166

BMI; body mass index, TLCobb’s; thoracolumbar Cobb’s angle

**Table 2:** Comparisons of thoracolumbar QCT and Cobb’s angle between Groups (n = 312)

Variables	Study group (n = 102)	Control group (n = 210)	p
QCT (mg/cm^3^)			
L1	64.20 ± 23.56	82.15 ± 24.86	0.000*
L2 BMD (mg/cm^3^) TLHCobb’s TLCobb’s DCTL	65.16 ± 25.44 70.18 ± 23.99 19.95 ± 4.53 12.74 ± 6.24 7.21 ± 2.10	85.16 ± 25.57 91.95 ± 25.06 18.96 ± 4.91 11.32 ± 6.45 7.64 ± 2.16	0.000* 0.000* 0.108 0.101 0.237

^*^p < 0.001, BMD was the mean of QCT-L1 and QCT-L2, DCTL was TLHCobb’s minus TLCobb’s

QCT; quantitative computed tomography, BMD; bone mineral density, TLHCobb’s; thoracolumbar hyperflexion Cobb’s angle, TLCobb’s; thoracolumbar Cobb’s angle, DCTL; the difference of Cobb’s angle of TLHCobb’s and TLCobb’s

Spinal OA Grade 1, 2 in the study group was significantly more prevalent than that in the control group, with OR = 1.43 (95% CI 0.74–2.78). DSN was found to be different between the two groups with OR = 2.34 (95% CI 1.38–4.00), and OPH was different from the study group to the control group with OR = 0.57 (95% CI 0.34–0.97) (Table 3).

**Table 3:** Comparisons of lumbar OA between groups (n = 312)

Variables	Study group (n = 102)	Control group (n = 210)	p
Spinal OA [n (%)]	88 (86.27)	171 (81.43)	0.000*
DSN	78 (76.47)	122 (58.10)	0.000*
OPH	69 (67.65)	165 (78.57)	0.000*

*p < 0.001

OA; osteoarthritis, DSN; disc narrowing, OPH; osteophytes

The Cobb’s angles of thoracolumbar spine were significantly different between the study and control groups after adjusting for age, BMI, and QCT values (Table 4).

**Table 4:** Comparisons of thoracolumbar Cobb’s angles between subgroups (n = 215)

Variables	Study group (n = 102)	Control group (n = 113)	p
Age (year) BMI (kg/m^2^)	66.47 ± 6.11 25.11 ± 2.48	64.67 ± 6.99 24.79 ± 2.39	0.063 0.302
QCT (mg/cm^3^)			
L1 L2 BMD (mg/cm^3^) TLHCobb’s TLCobb’s DCTL	64.20 ± 23.56 65.16 ± 25.44 64.68 ± 24.34 19.95 ± 4.53 12.74 ± 6.24 7.21 ± 2.10	67.20 ± 23.31 72.07 ± 26.76 69.63 ± 24.83 16.72 ± 4.77 7.80 ± 5.96 8.92 ± 2.05	0.429 0.088 0.185 0.000* 0.000* 0.000*

*p < 0.001, BMD was the mean of QCT-L1 and QCT-L2, DCTL was TLHCobb’s minus TLCobb’s

BMI; body mass index, QCT; quantitative computed tomography, BMD; bone mineral density, TLHCobb’s; thoracolumbar hyperflexion Cobb’s angle, TLCobb’s; thoracolumbar hyperflexion Cobb’s angle, DCTL; the difference of Cobb’s angle of TLHCobb’s and TLCobb’s

Prevalent number of vertebral compression fractures was found to be significantly associated with DCTL. The threshold value 8.7°of DCTL was determined by the ROC curve and Youden’s index (Fig. 6), with a sensitivity of 78. 4%, a specificity of 74.3%, an AUC of 0.783 (95% CI 0.613–0.953), and OR = 9.95 (95% CI 5.31–18.64). DCTL were classified into mild subgroup with DCTL ≥ 9.2°, moderate subgroup with DCTL from 9.2° to 7.5°, and severe subgroup with DCTL ≤ 7.5° (Table 5).

**Table 5:** Characteristics of subgroups by DCTL (n = 215)

Variables	Mild ≥ 9.2°	Moderate 9.2°-7.5°	Severe ≤ 7.5°	p
Prevalent fracture [n (%)] Age (year) BMI (kg/m^2^)	16/76 (21.05) 62.78 ± 4.01 24.16 ± 2.20	29/67 (43.28) 60.67 ± 5.75 25.78 ± 2.52	57/72 (79.17) 71.6.64 ± 5.26 27.87 ± 2.59	0.000* 0.000* 0.000*
QCT (mg/cm^3^)				
L1 L2 BMD (mg/cm^3^) TLHCobb’s TLCobb’s DCTL	74.77 ± 15.31 75.32 ± 17.61 77.06 ± 16.39 16.35 ± 3.45 6.27 ± 3.37 10.08 ± 0.56	78.16 ± 20.96 81.71 ± 26.69 79.93 ± 23.71 15.35 ± 4.73 6.77 ± 4.97 8.59 ± 0.41	44.75 ± 17.85 45.63 ± 16.59 45.19 ± 16.72 22.96 ± 2.32 17.37 ± 3.88 5.58 ± 1.94	0.000* 0.000* 0.000* 0.000* 0.000* 0.000*

*p < 0.001, BMD was the mean of QCT-L1 and QCT-L2, DCTL was TLHCobb’s minus TLCobb’s

BMI; body mass index, QCT; quantitative computed tomography, BMD; bone mineral density, TLHCobb’s; thoracolumbar hyperflexion Cobb’s angle, TLCobb’s; thoracolumbar hyperflexion Cobb’s angle, DCTL; the difference of Cobb’s angle of thoracolumbar segment

The fracture in both the moderate subgroup and the severe subgroup was significantly more prevalent than that in the mild subgroup, with OR = 3.84 (95% CI 1.80–8.20) and OR = 16.94 (95% CI 7.40–38.77), respectively.

## Discussion

Osteoporotic fractures are very common in postmenopausal women. Prevalence of the fracture reached 9.9% in a 9-year follow-up study [28]. Currently, the medical management for osteoporotic fractures in postmenopausal women focuses on how to prevent the occurrence of fractures [29, 30]. It has been accepted in previous studies that age, gender, low body mass index, previous fragile fracture, history of hip fracture in parents, glucocorticoid treatment, smoking history, excessive drinking, etiology of secondary osteoporosis and excessive spinal kyphosis are the long-term risk factors of osteoporotic fracture [24, 31–34]. However, few studies have noted the association of changes in spinal posture with the risk of fracture in osteoporotic spinal fractures. In our study, we found that the spine of most patients changed from neutral position to flexion position during low-energy spinal injury, as shown in Fig. 5. The findings suggested that this posture change during spinal injury may be a factor contributing to the increased risk of vertebral compression fracture in postmenopausal women.

Van der Jagt Willems et al. suggested that the elderly with thoracic hyperflexion posture are likely to fall in the next year [35]. Hyperflexion posture indicates that the ability of pace, balance and adjustment is reduced [36, 37]. Some studies shown that age-related flexion posture accelerates the degeneration of intervertebral disc [14, 38]. Due to the transformation of intervertebral disc from "liquid" to "solid," especially the anterior fibrous ring of intervertebral disc, the function of spinal physiological flexion and extension decreases [10, 12, 15]. Therefore, the dysfunction of spinal flexion and extension (We defined the decrease in the range of Cobb's angle from neutral position to flexion position as spinal flexion dysfunction) may accompany with the increased risk of falls in the elderly. On the other hand, the degeneration of intervertebral disc significantly changes the mechanical transmission mechanism of spine and changes the stress distribution of vertebral body, especially increases the stress in the anterior part of vertebral body [13, 15, 39]. These changes may lead to the decrease in disc cushioning capacity. As a result, the reduction in spinal cushioning capacity in low-energy injury may accompany with spinal flexion dysfunction. According to the above two reasons, spinal flexion dysfunction may increase the risk of vertebral compression fracture. In this study, it was found that thoracolumbar flexion dysfunction was significantly related with the risk of thoracolumbar vertebral compression fracture, as shown in Fig. 5 and Table 5, by which that conclusion was supported. Normally, hyperkyphotic posture involves the decreased motion of spine either as extension movement or as flexion movement from neutral position. In the present study, DCTL was found to decrease from 10.08° to 8.59° and to 5.58° in Table 5. In addition, DCTL was found in an decreasing trend while number of prevalent vertebral compression fractures was found in an increasing trend from 16 of 76 women (21.05%) to 29 of 67 (43.28%) and to 57 of 72 (79.17%). Therefore, if postmenopausal women with DCTL ≤ 8.7° (as a indicator of high risk of vertebral compression fractures) are found, necessary intervenes against future fractures can be expected.

The decrease in intervertebral disc height or DSN may increase spinal flexion dysfunction. Two cross-sectional and 11-year-followup prospective studies shown that DSN increases the risk of future vertebral fractures in postmenopausal women [16, 40]. The study presented that the DSN score of lumbar spine and thoracic spine (one item of the spinal OA grading scale) was related to vertebral fracture. Our study also showed that there was significant difference in lumbar DSN score between the two groups (*p* < 0.001). The relationship between DSN and DCTL was found with r = 0.893 (*p* < 0.001), and the fracture risk of patients with DSN was 2.34 times higher than that of patients without DSN.

Previous studies have shown that low BMD increases the risk of future fractures [41, 42]. In this study, there was a significant difference in BMD between the study group and the control group, with the difference reaching 20 mg/cm^3^. This result reach an agreement with the concept that low BMD leads to the increased risk of vertebral fracture. On the other hand, a prospective study of the Bone Microarchitecture International Consortium showed that microstructure defects in cortical and cancellous bone of the distal radius and distal tibia were independent predictors of fractures in the elderly [43]. In the present study, varying degrees of cavitation in the anterior part of thoracolumbar vertebral body, changes in trabecular structure, and decreased QCT values, around 1/3 to 1/4 lower than the middle or rear of the vertebral body were found in most subjects in the study group. Whether the changes of these bone microstructure can independently predict fractures in the elderly remains to be further explored.

Some caution is advised in the interpretation of these data. This study was conducted in postmenopausal women treated in a comprehensive tertiary hospital. The generalizability of this information to other populations is unknown. First, the small sample size may not be enough to evaluate the association between thoracolumbar vertebral compression fractures and DCTL. In the next step, multi-center study will be conducted to clarify the association. Second, this study focused on the thoracolumbar fracture in postmenopausal women, without including the other parts (lumbar spine, thoratic spine, humerus, hip, and wrist). Therefore, the study is limited. Third, 97 of 210 women in the control group were not included because of adjusting age, BMI, and QCT values; this may constitute a source of bias. Fourth, the association between the microstructure differences of anterior vertebral body and vertebral fractures between the study group and the control group could not be assessed in this study, which will be further explored in the following studies.

## Conclusions

Thoracolumbar vertebral compression fractures are significantly associated with DCTL in postmenopausal women. The risk of the fracture in postmenopausal women with DCTL ≤ 8.7° was 9.95 times higher than that with DCTL > 8.7°. Therefore, a DCTL ≤ 8.7° can be used as a high-risk marker of thoracolumbar vertebral compression fractures in postmenopausal women. While DCTL is not widely available, our findings may prompt expansion of the clinical use of DCTL and provide the clue to develop new models for fracture prediction.

## Data Availability

The datasets generated and/or analyzed in the current study are not publicly available due to the confidentiality policy of military hospital but are available from the corresponding author on reasonable request.
